# Medical education in Israel 2016: five medical schools in a period of transition

**DOI:** 10.1186/s13584-016-0104-5

**Published:** 2016-09-15

**Authors:** Shmuel Reis, Jacob Urkin, Rachel Nave, Rosalie Ber, Amitai Ziv, Orit Karnieli-Miller, Dafna Meitar, Peter Gilbey, Dror Mevorach

**Affiliations:** 1 Faculty Development Unit, Bar Ilan University Faculty of Medicine in the Galilee, Henrietta Szold 8 St, Safed, 13100 Israel; 2 Joyce and Irving Goldman Medical School of Ben-Gurion University of the Negev, Beer Sheva, Israel; 3 Ruth and Bruce Rappaport Faculty of Medicine, The Technion, Haifa, Israel; 4 Sheba Medical Center and Sackler Faculty of Medicine, Tel Aviv University, Tel Aviv, Israel; 5 The Hebrew University-Hadassah School of Medicine, Jerusalem, Israel

**Keywords:** Basic Medical Education, Israel, Medical Manpower, Medical Education Reform

## Abstract

**Abstract:**

We reviewed the existing programs for basic medical education (BME) in Israel as well as their output, since they are in a phase of reassessment and transition. The transition has been informed, in part, by evaluation in 2014 by an International Review Committee (IRC). The review is followed by an analysis of its implications as well as the emergent roadmap for the future.

The review documents a trend of modernizing, humanizing, and professionalizing Israeli medical education in general, and BME in particular, independently in each of the medical schools. Suggested improvements include an increased emphasis on interactive learner-centered rather than frontal teaching formats, clinical simulation, interprofessional training, and establishment of a national medical training forum for faculty development. In addition, collaboration should be enhanced between medical educators and health care providers, and among the medical schools themselves.

The five schools admitted about 730 Israeli students in 2015, doubling admissions from 2000. In 2014, the number of new licenses, including those awarded to Israeli international medical graduates (IMGs), surpassed for the first time in more than a decade the estimated need for 1100 new physicians annually. About 60 % of the licenses awarded in 2015 were to IMGs.

**Conclusions:**

Israeli BME is undergoing continuous positive changes, was supplied with a roadmap for even further improvement by the IRC, and has doubled its output of graduates. The numbers of both Israeli graduates and IMGs are higher than estimated previously and may address the historically projected physician shortage. However, it is not clear whether the majority of newly licensed physicians, who were trained abroad, have benefited from similar recent improvements in medical education similar to those benefiting graduates of the Israeli medical schools, nor is it certain that they will benefit from the further improvements that have recently been recommended for the Israeli medical schools.

Inspired by the IRC report, this overview of programs and the updated physician manpower data, we hope the synergy between all stakeholders is enhanced to address the combined medical education quality enhancement and output challenge.

## Introduction

In 2009, reflecting on the impending inauguration of a fifth medical school, a brief overview of Basic Medical Education (BME) in Israel was published in Medical Teacher [1]. During the relatively short time that has elapsed, marked changes in Israeli BME have been introduced and an extensive external expert evaluation has become available [2], allowing a fresh and critical view of the current status and future perspectives for Israeli BME. Changes in medical education are experienced globally with changes both in the prevailing educational paradigms (towards student centered, experiential instruction), and in healthcare realities (chronic diseases, ambulatory and prevention focus).

This paper begins with a brief overview of the Israeli medical system and a description of the five existing medical schools. The paper continues with an assessment of the state of BME in Israel in 2015-6 and its impact on the healthcare system, notably the issue of a physician shortage. The paper concludes with an attempt to critically evaluate Israeli BME and some thoughts for the future.

The authors are senior members of the five medical schools, who researched their institutions in order to supply up-to-date and precise reports according to a common, pre-designed framework. However, their reports represent their perspectives and are not always officially endorsed by their institutions.

### Healthcare in Israel

Since 1995, Israelis have been covered by a national universal health insurance law under which all permanent residents are insured in one of four competing Health Funds [3]. Life expectancy, infant mortality and many other health care indices in Israel are similar or better than average European indices [4]. Health care expenditure was 8 % of GNP in 2014, below the Organization for Economic Cooperation and Development (OECD) average of 9.3 % [5]. In 2016, the major challenges of the health care system include sustainability, physician supply, and lack of a primary care focus. For more extensive description of the Israeli system see also [1, 2, 6].

### Israeli Medical Education

#### BME

Currently five medical schools exist in Israel, admitting about 730 Israeli students for the 2015–2016 academic year in the various programs (Table 1); this is double the level of enrollment in 2000.

**Table 1:** The number of students in the different programs in Israeli medical schools

School	Israeli MD students (per year, excluding English-language programs)	Foreign- English-language programs	Israelis returning after 3 years abroad	Graduate, entry level
Rappaport, Haifa	130	45	8	7
Sackler, Tel Aviv	185	75	–	65
Hebrew Univ, Jerusalem	180	0	–	–
Goldman, Beer Sheva	130	50	–	–
Bar Ilan, Safed	105	–	41	64
Total (2015)	730	170	49	136

In addition to the Hebrew-language Israeli programs, there are English-language programs in three Israeli universities. These recruited a total of about 170 students in 2015, mostly US citizens who return to their native country upon graduation and are not licensed to practice medicine in Israel, thus having minimal or no impact on Israeli physician numbers.

Overall, the current level of enrollment is still short of the need for 1000–1200 new physicians per year that was predicted by three official committees from 2002–2010^1^ [7–11]. This figure was set to achieve a physician to population ratio at 2.9 per 1000, while accommodating both retirement and population growth, and assuming licensure of 285 IMGs per year.

In the past, the majority of Israeli physicians trained abroad; they were either new immigrants who did their training prior to immigration or Israelis who left Israel temporarily to train abroad. For about a decade until 2013 the numbers of locally and abroad trained newly licensed physicians were about equal, and in the last 2 years the proportion of the IMGs is growing (Table 2). Most of these are returning Israelis. This is why the need for domestically educated physicians may be less than predicted previously as the number of Israelis who graduate medical schools elsewhere (International Medical Graduates-IMGs) has also increased substantially. Indeed, licenses were awarded to 537 graduates of Israeli schools in 2014 and 485 in 2015, compared to 324 in 2010 and 360 in 2011, as well as to 548 IMGs in 2014 and 774 in 2015, compared with 222 in 2010 and 284 in 2011 [12]. These figures compel a revisit of the predicted physician shortage (Table 2).

**Table 2:** Medical licenses granted

	2005	2006	2007	2008	2009	2010	2011	2012	2013	2014	2015
Israeli Grads	304	300	310	322	361	324	360	391	418	537	485
IMGs	297	285	238	294	316	222	284	527	600	548	774
Total	601	585	548	616	777	722	899	918	1018	1185	1259
Difference from estimated requirement (1100 a year)	-499	-515	-552	-484	-323	-378	-201	-182	-82	85	159

*IMGs* International Medical Graduates

The standard Israeli BME program includes 6 years of medical school at the undergraduate level, and an additional year of rotating internship. Two 4-year, graduate-level entry programs exist, one in The Sackler Faculty of Medicine of Tel Aviv University and one in the Faculty of Medicine in the Galilee of Bar Ilan University. Bar Ilan also offers a 3-year clinical program for 40–50 Israelis returning after 3 years of medical school abroad.

The curricula at three of the four veteran schools, The Hebrew University-Hadassah School of Medicine in Jerusalem, Sackler Faculty of Medicine of Tel Aviv University, and the Rappaport Faculty of Medicine of the Technion in Haifa, have historically been very traditional, while the medical school of Ben Gurion University in Beer Sheba, now known as the Goldman School of Medicine, introduced an innovative community-oriented, integrative, student centered program with early clinical exposure from the start [13]. However, all four veteran medical schools have reviewed their curricula in the past two decades, in an attempt to integrate selected modern educational paradigms [1].

BME in Israel is regulated by the Council for Higher Education (CHE), an agency of the Ministry of Education, which allocates budgets, determines national academic policies, and is charged with quality assurance of higher education in general, including medical education. This agency is also the body that authorized the establishment of a fifth medical school and commissioned an International Review Committee (IRC) as part of a review process [2]. A formal periodic review of medical schools was introduced by the CHE in 2000 and repeated in 2007. In June 2014, an IRC submitted a new report following visits to all Israeli medical schools [14].

### Licensure

There is no statutory medical licensing examination in Israel for Israelis who are graduates of any of the five Israeli medical schools. Uniform exit examinations in five disciplines were introduced by the deans of Israeli medical schools about 20 years ago [15], and all medical schools participate. A license to practice medicine is granted by the Ministry of Health to graduates of Israeli medical schools after completion of a 1-year internship. In general, Israeli graduates have an excellent reputation internationally [1, 16]. IMGs are required to take a licensing examination before they can seek employment or apply for residency training [17]. The number of IMGs who took the licensing examinations from 2010–2014 varied from 489 to 608 candidates annually [12].

### Present State of the five Medical Schools

In the Appendix and in Table 3, the authors describe their respective institutions in order to allow a comprehensive view of the present state of BME in Israel, thus supplying the necessary context for the ensuing analysis.

**Table 3:** Key features of medical education in the five Israeli medical schools

	Jerusalem	Tel Aviv	Haifa	Beer Sheva	Safed
When established	1949	1964	1970	1974	2011
Types of programs	6 year with 4 patterns (regular, Military Medicine, research oriented, community oriented)	6 year 4 year Foreign students	6 year Foreign students	6 year Foreign students	3 year 4 year
Admissions process^a^	MMI (in house)	MMI (MSR)	MMI (in house under MSR supervision)	Computerized screening and interview	MMI (MSR)
Type of curriculum^b^	Traditional until Oct-2016, when a reform was launched	Originally traditional, later reformed	Originally traditional, later reformed	Originally innovative, further reformed in a number of cycles	Innovative from the school’s establishment
Faculty development	Yes	Yes	Yes	Yes	Yes
Teaching Methods: - Number of small group/self-directed courses - Lecture-based courses - Team-based learning	At least 1 Yes Not yet	At least 1 Yes Not yet	At least 1 Yes Not yet	At least 2 Yes Yes	At least 3 Yes Yes
Physical infrastructure	Adequate (but more small group space needed)	Adequate (but more small group space needed)	Limited	Adequate	Limited
Humanities requirement	Yes	Yes	Yes	Yes	Yes
Methods for student assessment	Traditional – predominantly MCQ	Mixed – MCQ+ OSCE + other	Mixed – MCQ along with innovative modalities	Mixed, with extensive use of OSCE	Mixed
Weight of teaching performance in promotion decisions	Minimal,except in a teacher track (5 % of promotions)	minimal	minimal	substantial	minimal
Service learning	Required	Required	Elective	Elective	Required
Recently added courses	Humanities	Professionalism emphasis, longitudinal strands	Literature and medicine, genomics and medicine, entrepreneurship and medical technologies	Interprofessional education (IPE) and some of the humanities courses.	The school is new, so full curriculum is new. ETGAR course – transition between hospital and community \care.
Prizes & awards for teaching	Yes	Yes	Yes	Yes	Yes
Medical education unit	Center	Department	Unit	Center	Distributed
Future plans	Adding tracts, Significant community exposure increase	Physician self-care, gender medicine, and enhanced assessment of professionalism	Increase in the staff of the medical education unit	Simulation center, strategic plan to redesign the curriculum	Expand interprofessional education and increase the use of simulation.

^a^Multiple Mini Interviews (MMI) is an admission method based on multiple short observed simulated interactions and interviews [18]. The Israeli version was initially developed in the national simulation center (MSR) and subsequently adopted for in house production by two of the schools (one, Haifa, does an identical version to MSR)

^b^A traditional curriculum is one that has a disciplinary, teacher and lecture basis. An innovative curriculum is one that is integrative, with a student and small group/ self-directed learning basis

As indicated in Table 3, all five schools have recently undertaken significant steps to update and upgrade medical education. The medical school in Jerusalem is embarking on its first comprehensive, integrative curricular reform, and each of the other older three medical schools has moved ahead in several key areas in recent years. The medical school in Safed, which was established in 2011, adopted an innovative curriculum from the start.

While there are differences among the medical schools in their admissions processes, teaching methods and student assessment models, there is also a common trend toward considering non-cognitive attributes in admissions, moving towards more interactive and small group teaching, and adding performance and computer based testing to the prevalent MCQ based examinations. Three of the five schools consider their teaching spaces wanting. Sackler enjoys access to two simulation centers in its affiliated hospitals, while the four other schools are in various stages of incorporating simulation as part of their infrastructure. Where the consideration of teaching excellence, scholarship and leadership in promotion and tenure is concerned, Beer Sheva is the most progressive in including it as a significant promotion criterion, and Jerusalem has recently added a modest teacher promotion tract. All five schools include humanities in their curricula, and all are moving in the direction of more inter-professional education, a clearer focus on professionalism, and strengthening of their medical education teams.

### How appropriate is Israeli BME?

During the past 5 years, as described above and in Table 3, the trend of modernizing, humanizing, and professionalizing Israeli medical education in general, and BME in particular, has materialized independently in each of the medical schools. Israeli medical graduates’ grades on international examinations (i.e. USMLE part 2) are comparable to those for graduates of American medical schools (the same average), and markedly higher than graduates from other OECD member countries such as the UK [16]. This may support the assertion that BME in Israel is effective, in the sense that medical schools are producing academically competitive graduates. However, since only a self-selected (albeit large) group of Israeli graduates takes the USMLE, it is unclear to what extent scores for this subset of students are representative of the graduating classes as a whole. The IRC, recently commissioned to evaluate Israeli BME, reported that there is still room for improvement. The main domains deemed to be in need of further reform are discussed in the next section.

### Thoughts for the future

This section discusses the concerns and issues that characterize Israeli BME in 2015,-6 based on the IRC recommendations [14] and the authors’ experiences, and supplies a roadmap for further improvement.

I. Admissions: Two schools (Sackler-Tel Aviv University and Bar Ilan-Safed) collaborate on an admissions procedure that incorporates evaluation of non-cognitive attributes based on an MMI process. Interviews are conducted at the national simulation center (MSR) [18]. The Hebrew University have adopted a modified Multiple Mini Interviews (MMI) system, which they presently run in-house, Rappaort runs an i d entical to MSR procedure in-house, While Goldman-Ben Gurion continues to rely on a more elaborate interview system. An investigation of the predictive validity of the various admissions procedures appears to be warranted. Evaluations are underway at the Hebrew University-Hadassah Medical School [19] and in advanced planning at Bar Ilan.
II. Exit Examinations: As already mentioned, about 20 years ago the deans of the medical faculties decided to hold an integrative joint exit multiple choice questions examination for all five schools [15]. Recently, a task force selected by the deans visited the US-based National Board of Medical Examiners (NBME), which led to proposals to adopt a version of the USMLE; however, the plan was rejected. The IRC has recommended introduction of additional assessment methods, e.g., an Objective Structured Clinical Examination (OSCE), to ensure that the desired threshold of clinical competence has been achieved by students [14].
III. Collaboration Between Medical Educators and Health-Care Providers: In 2007, and again in 2014, the IRC recommended that “the Council for Higher Education (CHE) or another national agency play a major role in designing and implementing a coordinated plan for all the resources needed for effective “medical and other health professions education” based on “comprehensive health care and physician workforce planning” [14]. We fully endorse this recommendation.
IV. Medical Education: There is an apparent lack of expertise in the science of education as applied to medical education in Israel. Only a handful of academic professionals have formal credentials in medical education. The IRC recommends building medical education centers to provide the needed expertise [14]. The authors share this sentiment, and most medical schools include strengthening their medical education operation in their future plans. One recent positive development, recommended also by the IRC and supported by the deans of medicine, has been the establishment of the Israeli Society for Medical Education (HEALER).
V. Faculty Development: The IRC recommends that the CHE should encourage each faculty of medicine or health sciences to create a portfolio of faculty development and remediation activities and demonstrate that faculty performance is improving in all domains of medical education [14]. HEALER has already made this issue a priority and is initiating nationwide faculty development activities.
VI. Teaching and Assessment Methods: Current medical education paradigms, as the IRC, recommend a reduction in dependence on frontal lectures, providing more opportunities for interactive learner-centered small group and discussion formats (problem-based and team-based learning methods, for example), and for learning in clinical settings, even early in the medical curriculum. Students should be provided with opportunities for more active involvement in patient care activities, in both inpatient and ambulatory settings [14]. In the same vein, reduced dependence on multiple choice examination formats for student assessment is deemed desirable. Increased use of performance-based assessments using faculty observation, patient instructors, or other clinical simulation methods as appropriate, is recommended. Finally, wherever individual students or small groups have contact with a faculty member over a period of a few weeks or more, the student should receive formal, written formative performance feedback, which should become a part of the student’s educational record [14].
VII. Simulation: The IRC found that each of the schools is underutilizing clinical simulation methods for both teaching and performance assessment. Further resource development, from small-task trainers to whole-body simulators, and including patient-instructor/standardized patient methods, is needed. This is another area in which nationwide collaboration, utilizing resources such as the Israel Medical Simulation Center (MSR) [18], may be helpful [14].
VIII. HEALER: The IRC calls for nurturing, supporting and sustaining HEALER, the new organization dedicated to scholarship in medical education, so that it can help in in developing a national infrastructure for medical education [14]. The Israeli Society for Medical Education (HEALER) aims to fill this role, and to foster collaboration among the five faculties of medicine as well as the educational bodies of other providers of health profession education, to establish a resource center, and to promote several specific interest groups.
IX. Interprofessional Education (IPE): In the absence of a significant amount of team-based learning and interprofessional education (which are now much more available in other developed countries), there is little educational infrastructure to prepare graduates for working in teams. IPE needs to become prevalent beyond the Goldman Medical School [14].
X. Funding for Medical Education: The IRC states that Israel should specifically reconsider the funding model for medical education, including all the funding of those involved in physician education at all levels, and not only at the undergraduate level. This should involve the Ministry of Education through its higher education finance bodies (BME & some residency positions), the Ministry of Health (for the internship year & residency positions), healthcare providers and institutions (mostly for residency positions), the Ministry of Absorption (for new immigrants training) and additional bodies. Failure to do this could lead to ineffective or inefficient programs for the education of the physician workforce needed for the 21^st^ century [14].
XI. Shortage of physicians, International Medical Graduates: If the trend s reported above, about the changing numbers and composition of newly licensed physicians in Israel continue, then up to two thirds of newly qualified physicians in Israel will be trained in countries where some of what the Israeli programs (and the IRC) see as basic may be absent (i.e. communication skills training, ethics, EBM, to name a few). It behooves the relevant stakeholders to further study the implications and consider realigning policies such as the licensing requirements (i.e. addressing missing domains as above), and the educational imperatives (i.e. educational oversight of the internship year, linking needs with their acquisition evaluation), as well as manpower planning and its consequences (i.e. growing lack of residency positions).

## Conclusions

This paper has presented an up-to-date description and evaluation of the Israeli medical education system and highlighted several important developments and challenges. First, a change is noted in the numbers of both Israeli graduates and IMGs, which may, on one hand, eventually address the present physician shortage, and on the other hand, adds to the concerns mentioned above. The quality of Israeli medical school graduates appears to be internationally competitive, as judged by graduates’ performance on standardized international examinations [16]. At the same time, the recent IRC report, as well as the authors’ reports and analysis, outline a roadmap for further improvement [14]. The salient features that require strengthening, according to the IRC, include “enhancing the coordination and efficiency of medical education across the continuum of education and training, and re-examination of the financing of medical education” [14].

For the authors, the importance of development of policies, teaching, and assessment methods, as well as an expertise in the science of medical education, are additional building blocks for future positive change. However, the possibility that 60 % of newly licensed physicians in Israel will not benefit from this hoped-for improvement is a cause for concern.

Inspired by the IRC report [14] and this analysis, we hope for expansion and deepening of the collaboration between the five Israeli medical schools. The existing collaboration is focused on exit examinations, the deans’ forum, and support for the establishment of HEALER. Further collaboration is embodied in the writing of this paper. We hope for future enhancement of this collaboration where each of the schools as well as HEALER contributes. As each school has its unique strengths and particular innovations, amply described in the Appendix and Table 3, these can be shared and serve as a common asset to others as needed. In addition, we hope that better integration of all stakeholders and other institutions relating to education in the health professions will materialize in the near future.

With this cooperation, we hope that continuing improvement in the quality and effectiveness of Israeli medical education will be fostered, resulting in better health for all within the country and hopefully the region beyond our national borders.

### Policy implications

- The recent addition of a fifth medical school in Israel and an international audit of Israeli medical education provide a rare opportunity for a detailed review of Israeli basic medical education.
- While the quality of Israeli medical graduates as measured by the performance of a sample taking the USMLE is competitive, room for improvement exists and is analyzed in detail.
- Enhancing the coordination and efficiency of medical education across the continuum of education and training, and re-examination of financing mechanisms for medical education, as well as increased collaboration in development of policies, teaching, and faculty assessment methods, and building national expertise in the science of medical education, are building blocks for future positive change.
- The present shortage of physicians may be corrected if present levels of student recruitment and returning IMGs persist. However, this will also mean that it is not clear whether two thirds of newly licensed physicians in Israel will benefit from improvements in Basic Medical Education recommended in this paper.

## Appendix: Description of the 5 Israeli medical schools

### The Hebrew University-Hadassah School of Medicine, Jerusalem

The Hebrew University together with the Hadassah Medical Organization established, in 1949, the first medical school in Israel. Today the Faculty of Medicine encompasses schools of Medicine (>1000 students), Pharmacy (400 students), Public Health and Community Medicine (120 students), Nursing (800 students), Occupational Therapy (240 students), health professions programs (250 students), and Programs for Advanced Studies (650 Masters and Doctoral level students). The School of Dental Medicine (500 students in a D.M.D. program) is organized as a separate faculty, but there is overlap in the preclinical curriculum between the medical and dental Schools.

The School of Medicine offers two 6-year MD programs, the “regular” 6-year program and the recently introduced (2009) 6-year Military Medicine program. The number of medical students increased from around 640 m in 2008–2009 to over 1000 medical students in 2012–2015, due to the new Military Medicine program; this presented a major challenge from the work load perspective. Teaching is delivered by 1000 teachers, including 108 pre-clinicians, with the remainder clinicians; 400 teachers hold academic appointments

**Admissions:** About 10 % of applicants are admitted to the Medical School. Students are selected first by their matriculation and psychometrics scores and subsequently by a procedure known as MIRKAM that is based on a multiple mini-interview (MMI) procedure and emphasizes candidates’ non-cognitive attributes [18]. MIRKAM resembles the MOR procedure (MOR is a Hebrew acronym for ‘selection for Medicine’) that was conducted in three other medical schools in Israel.

**Curriculum**: Until recently, the curriculum was based on a traditional 3-year of preclinical studies followed by 3 years of clinical clerkships, with some innovations (to be described further below) that were added in the last decade. An advanced teaching program, based on an integrated medical curriculum that has been in planning since 2013, was recently approved by the Standing Committee and the Senate of the Hebrew University, and will be implemented beginning in the coming 2016-7 academic year.

**Faculty development**: There are multiple opportunities for faculty development, both in-house and through the University’s teaching and learning support unit for faculty development. In preparation for the curricular reform, additional opportunities tailored specifically for teachers of the renewed integrated science curriculum have been developed and they are introduced through the remainder of 2016. In addition, an online teacher’s empowerment course is was launched in the summer of 2016.

**Teaching methods**: Many preclinical courses that were taught by traditional frontal lectures with little active learning or student engagement have been gradually modified using approaches such as small groups, problem-based learning (PBL), and larger groups for team-based learning (TBL); the goal has been to increase student involvement in the teaching process. Teaching methods in the clinical unit clerkships emphasize clinical skills and focus primarily on bedside teaching in small groups, personal mentoring, and case discussions. There has been a significant increase in the proportion of teaching in ambulatory settings, including the integration of community-based clinics during both the first year and the advanced years of medical school.

**Infrastructure**: The focus on small group learning was accompanied by the construction of state-of-the-art small group suites. Plans are in place to double the number of such premises. Additional learning spaces to support the curricular reform are also in advanced stages of planning. The University’s medical library provides teaching materials, a wide range of electronic teaching aids and calm spaces for individual and small group studying. Most of the preclinical courses are conducted in modern laboratories, computer classrooms, and a large anatomy dissection facility. Histology and pathology teaching is completely computerized.

**Humanities**: An example of curricular innovations over the last decade is the “Man and Medicine” program, which emphasizes communication skills and professionalism as well as the ethical and sociocultural aspects of health and illness. Students also study the patient experience by visiting patients at home, accompanying patients through interactions with the medical team, and writing a reflective journal on their experiences. This program was initiated in years 1–3 and was recently expanded into the clinical years with workshops such as breaking bad news and other clinically relevant subjects.

**Assessment**: All courses in the Medical School require passing a written examination, usually in the multiple choice question (MCQ) format. Upon graduation, all students are required to complete the uniform examination for all Israeli medical school graduates as well as an additional local examination in clinical skills. Plans to introduce a comprehensive preclinical examination (equivalent to the U.S. Medical Licensing Examination [USMLE] step 1), and at least one comprehensive objective structured clinical examination (OSCE) at the end of the fifth year are under consideration. Alternative assessment methods are used in the “Man and Medicine” course.

**Promotion & Tenure:** Academic appointments and promotions are primarily based on traditional academic criteria (i.e. publications, grant support, national and international scientific standing, and teaching excellence). A new teaching-based track was introduced 2 years ago in which promotion and tenure is evaluated mainly on the teaching activities, leadership and scholarship of the candidates.

**Service learning:** The Man and Medicine course includes learning units linked to both hospitalized patients (shadowing patients in the hospital or assisting in patient navigation within the in-patient system) and community based and voluntary organizations that serve for example the disabled, mentally handicapped, and persons special needs.

**Recently added courses:** In recent years, several courses have been introduced within the preclinical curriculum with the aim of providing a better bridge between the preclinical and clinical worlds. They also seek to introduce students to the real life of the doctor at an earlier stage of medical education. The courses include medical law, family medicine, evidence-based medicine, medical humanities, and introduction to public health.

**Prizes & awards**: More than 50 prizes and awards for teaching as well as research excellence are awarded by the Faculty. These are publicized widely and are presented during a prestigious annual gathering.

**The Center for Medical Education:** The Center was established with a focus on the development of advanced teaching methods, including integrative interactive computerized courses, the OSCE, multi-clinical station examination (MCSE), and teaching skill enhancement tools. It is staffed by an MD MHPE medical educator, and a full-time administrator and secretary. Two emeritus professors with a special interest and expertise in e-learning safety and medical education are also a member of the center staff. The Committee for Medical Education, which acts as the driving force for incorporating changes in the content and teaching methods, serves the Center’s steering committee.

**Future plans:** In October 2016, a comprehensive curriculum reform for the Hebrew University-Hadassah Medical School will begin in the first-year of studies with an integrated science curriculum supported by longitudinal strands. This transformation is accompanied by a host of faculty development and quality assurance steps as well as efforts to renovate teaching and assessment methods as described. Among other changes, this program will enable students to select one out four tracks during their clinical years: the “regular” program; an enhanced research program including the possibility of obtaining a PhD (MD-PhD) or MSc (in basic science, public health, or health management); the Military Medicine program; and the community medicine-focused program. These tracks will open new possibilities for some students with an interest in a career development along a specific track while keeping a common obligatory core of clinicians.

### Sackler Faculty of Medicine, Tel Aviv University, Tel Aviv

The Sackler Faculty of Medicine was founded in 1964. The student body includes 750 Israeli students in the 6-year M.D. program, 260 Israeli students pursuing an M.D. degree in a 4-year graduate entry program, and 300 American and Canadian students enrolled in a 4-year M.D. program chartered by the State of New York. In addition, approximately 200 students study dental medicine in a D.M.D. program and 2000 students are enrolled in other health professions programs. In addition, Sackler’s Graduate School for Advanced Studies trains approximately 800 masters and doctoral level students, including an MD-PhD track. The School of Medicine’s academic staff includes 74 preclinical staff members and an additional 840 senior clinical staff members (Lecturer and above).

**Admissions**: A major reform, initiated about 10 years ago, introduced humanistic and communication skills criteria for student selection in addition to academic scores and achievements [18]. This led to the development of the MOR admission procedure within the National Simulation Center (MSR) that includes a MMI process and challenging communication simulations.

**Curriculum**: Sackler’s curricular reform occurred in 1998–1999, and its main features were described previously [1]. In recent years, additional changes were introduced including implementation of a competency-based curriculum for the preclinical years, public health and epidemiology studies, and a longitudinal mandatory course integrated into preclinical and clinical years focused on professional identity formation. More elective clerkships were added to the clinical years and several surgical subspecialties were merged with their medical counterparts.

**Faculty development**: Multiple opportunities for faculty development exist through the University and the Faculty’s teaching promotion and assessment unit. The unit developed simulative, hands-on workshops focused on promoting bedside teaching skills for tutors and clinical instructors, preclinical teaching skills, and improvement of exam quality.

The Department of Medical Education holds two annual events focused on enrichment of faculty skills in facilitation, and teaching communication skills and professionalism. Over the past 5 years, half of the medical education department faculty has participated in a year-long program to foster group facilitation skills.

**Teaching methods**: Various types of teaching and learning modalities are integrated, with increasing focus on self-directed learning through digital modalities, case-based learning (CBL), and small-group exercises. The use of simulation is partially facilitated by the availability of two simulation centers in Tel Aviv University-affiliated institutions. Reflective practice is implemented and encouraged. During the preclinical and clinical years, students are required to submit multiple reflective journals centered on professional identity formation, communication skills, and professionalism. Small-group facilitators provide personal feedback on these reflection and writing tasks.

**Infrastructure**: In the preclinical years, most studies are in the Sackler Building, which houses laboratories, teaching labs, computer classrooms, an anatomy dissection laboratory, and 24 lecture halls and classrooms. More small-group rooms are needed, as well as on-campus simulation space.

**Humanities/Humanism**: Sackler’s Department of Medical Education has introduced several innovative curricular initiatives throughout the years, including a theoretical course about human aspects in medicine and a mandatory, bi-weekly, small-group course facilitated by a physician-facilitator that supports professional development. This course includes early clinical exposure as well as year-long projects focused on building therapeutic relationships and learning how patients and families cope with illness. Educational units that incorporate simulation, videotaping, and feedback on communication skills are integrated into this curriculum. Besides basic communication skills, training includes more challenging skills such as shared decision making [20] and breaking bad news [21].

**Assessment**: Ongoing assessment throughout the years is based primarily on MCQ examinations. Professional development (including reflection capabilities and communication skills) is assessed by small-group physician-facilitators. The clinical years also include formal closed-end evaluations of cognitive skills and professional behavior. Currently a new professionalism assessment questionnaire is being integrated.

**Promotion & Tenure:** Academic advancement is based primarily on (1) publications in leading scientific journals, (2) presentations at international conferences, and (3) obtaining competitive funding for research. In addition, teaching experience and skills are required, with student online evaluation forming part of the assessment process.

**Service learning**: All students are required to do service learning. During the second and third years, each student has a 2–4 h bi-weekly meeting with a person or family living with a physical or mental disability. In addition, students are invited to enroll in two elective classes initiated by the medical students. One focuses on providing social and medical care for disenfranchised populations by visiting different care settings and learning about the population and the need from various care providers and from the people themselves; the other new initiative focuses on developing a project to help disadvantaged populations.

**Recently added courses**: In recent years, there has been a focus on developing “longitudinal” structures of several courses, including imaging, epidemiology/statistics/research, and behavioral sciences and professionalism. Additional courses recently integrated into the medical curriculum include evidence-based medicine, patient safety, emergency medicine, and health promotion.

**Prizes**: Teaching excellence is recognized by the Rector with awards to outstanding teachers and junior faculty. Furthermore a “list of Best Instructors of the Medical School” is published each year on Faculty and affiliated Medical Center websites. Various internal grants are given every year by the faculty to excellent research proposals.

**The Department of Medical Education:** The Department includes about 100 part-time faculty from various fields of medicine, who are primarily small-group facilitators. The Department Chair is supported by a senior physician-educator who serves as head of educational programs, and another tenured senior faculty member who heads social science research. Additional part-time scholars from the social and behavioral sciences are also members of the department.

**Future plans**: The medical faculty plans to integrate units on physician self-care, gender medicine, and enhanced assessment of professionalism into the curriculum during the pre-clinical and clinical years.

### Ruth and Bruce Rappaport Faculty of Medicine, The Technion, Haifa

The Faculty of Medicine was established in 1969–70 and became affiliated with the Technion-Israel’s Institute of Tecnology in 1973. The Faculty includes a School of Postgraduate Medical Sciences whose MSc and PhD candidates are actively involved with faculty researchers in ongoing scientific and medical research. Special programs for outstanding students combining multidisciplinary education and research include MD-PhD and MD-Engineering programs, as well as the MD-Law program in collaboration with the University of Haifa. In addition, the faculty grants a BSc in laboratory medicine and collaborates with the University of Haifa in the Occupational Therapy Program. The Faculty staff is comprised of 450 educators, including 45 preclinical scientists. The student body is comprised of roughly 635 Israeli medical students (115 admitted in 2015-6 for the first year class, with about 15 additional students joining later in the program, either after completing a BSc or returning from 3 years of studying medicine abroad, thus 128 students graduated in 2016), 165 foreign students in an English-language program, and 200 graduate Medical Sciences students.

**Admission:** Criteria for admission focus on matriculation and psychometric grades in addition to a MOR process identical to one carried out in MSR (see above),now administered in –house (after being conducted in MSR before)

**Curriculum**: The objective is to educate physicians with a strong background in science and technology and to develop their potential as excellent professionals, whether clinicians or researchers. The first 3 years are devoted to courses in the basic sciences as well as a 3-year longitudinal course, “Being a Doctor - Exposure to the Medical System”. In this course, run weekly, small groups of students with a single tutor are exposed to the daily activities of physicians in different settings, and are confronted with various social, medical, and ethical dilemmas. The “integrative course” bridges between the preclinical and clinical studies and takes place 5 days a week for two trimesters in the fourth year of studies. Students are exposed to the pathophysiology and clinical manifestations of diseases affecting the various body systems. This course is run in parallel with the Introduction to Clinical Medicine-Physical Diagnosis course, and passing it satisfactorily is a prerequisite for beginning the first clinical clerkship. The third trimester of the fourth year is devoted to this first clerkship, which is in internal medicine for all students. The last 2 years are devoted to additional clinical clerkships. All students participate in Advance Trauma Life Support (ATLS), Pediatric Advanced Life Support (PALS) and medical management workshops. During the sixth year, all students serve as “mini-interns” during clerkships in internal medicine and paediatrics.

**Faculty development**: The Faculty of Medicine offers a range of faculty development activities, including workshops for improving the quality and rigor of MCQ, teaching skill enhancement (e.g., providing effective feedback and assessment of clinical competence), and preparation for tutors in the various clerkships and the “Being a Doctor” course. In addition, an annual faculty development program (FDP) was established 6 years ago [22], which consists of 7 monthly workshops based on small group active learning, online forums, and a continuous appraisal process.

**Teaching methods**: The full range of modern methods are integrated into the curriculum, including lectures, small groups, E-learning, modified PBL, and CBL.

**Infrastructure**: The Rappaport facility is the center for medical education. It houses classrooms, an anatomy laboratory, a simulation center, teaching and research laboratories, as well as 12 small-group teaching spaces. In addition we use our affiliated hospitals simulations’ facilities options in our clinical teaching environment.

**Humanities**: The “being a doctor” 3 year longitudinal course has been described above. To increase student awareness of ethical issues and medico-legal dilemmas, the Faculty has organized an annual ‘Patient-doctor relationship day’ with student participation in both preparation and content, for the past 20 years. Topics covered over the last 5 years include the patient-doctor relationship and truth telling, alternative medicine, physician burnout, information technology, and the challenging patient. In addition, several courses focus on humanities such as “literature and Medicine”, “medicine and the holocaust” and “The Genome and ethical dilemmas”.

**Assessment:** The pre-clinical years are characterized mainly by frontal study, hence most of the examinations are MCQ, as well as laboratory reports and occasionally seminar presentations.

**Virtual Patients Examination (Computerized exam)** [23] This examination is conducted at the end of the introduction to clinical medicine, and at the conclusion of the internal medicine clerkship in year 4. We have developed an internet-based virtual patient system designed both for practice and evaluation of medical students.

**Multi Clinical Stations Examination (MCSE) -** This examination is conducted as a final oral examination at the end of the Pediatrics, surgery, and internal medicine clerkships. The goal of the MSCE is assessing clinical reasoning using independent assessors for each examination station.

**Comprehensive Integrative puzzle (CIP)** assessment [24]. We use the CIP assessment tool in the 4th year Integrative course described above. The goal of this examination is to match different diagnoses with the physical examination findings, laboratory test results, imaging findings and proposed treatments. The format of this examination is a grid, comparable to a jigsaw puzzle.

**Reflection as a formative and summative assessment tool:** In the course “being a doctor” the students provide written structured reflections about each and every encounter.

**Promotion & Tenure**: Academic advancement is based at the TECHNION on publications (publish or perish) and reception of research grants. However, no candidate is considered without a teaching portfolio that includes student evaluation of teaching effectiveness. The main tool for student assessments is an evaluation questionnaire.

**Service Learning:** Although participation in community programs as part of the learning process is not obligatory, students serve in a variety of voluntary capacities, such as teaching challenging pupils in the community (PERACH) and walking guide dogs for the blind. In addition the students themselves have initiated various service activities including activities designed to bridge the multi culture student population.

**Recently added courses**: Learning units that focus on literature and medicine, genomics and medicine, entrepreneurship and medical technologies have been added to the curriculum in recent years.

**Prizes & awards:** Once a year the Dean organizes a festive evening for honoring excellence in teaching. These teachers are chosen according to students’ assessment of teaching. In addition we honor excellence of clinical departments. In addition there is a Professor Gideon Alroy prize, chosen by the dean’s committee, for outstanding activity in medical education and a Professor Jacob Green prize, chosen by the students, for outstanding teaching. In addition there are Technion awards for excellence in teaching, i.e. The Jacknow award that are occasionally bestowed on medical school teaching staff.

**The Committee for Teaching Assessment:** The role of this Committee is Quality Control of Teaching, and includes collecting information from student evaluations and peer review to monitor and improve the quality of teaching. It operates independently of the medical education committee.

**The Medical Education Unit:** The Unit focuses on teaching, teachers and students. promoting diverse tools for teaching and assessment of clinical learning and teaching into the curriculum, leading faculty development programs and activities to enhance our students’ awareness of various aspects of the doctor-patient relationship (e.g. ethical dilemmas and communication), and conducting conferences and workshops focused on challenges in clinical teaching in the 21^st^ century. The Unit’s staff includes: (1) a full-time Ph.D. who heads programs in teaching assessment and promotion; (2) a part-time clinical faculty member who focuses primarily on communication and developing students’ clinical competence and professionalism; (3) a group of preclinical and clinical teachers who comprise the Committee for Medical Education that meets once a month. Students participate in all committee activities, and (4) an ad hoc committee for the annual ‘patient-doctor relationship day’.

**Future plans:** Key short-term objectives include a major increase in the staff of the medical education unit, opening new courses focused on humanities and physician professional competence (i.e. Mindfulness, Multiprofessional team based work, telemedicine among others), and formalizing students’ community service activities.

### Joyce and Irving Goldman Medical School of Ben Gurion University of the Negev, Beer Sheva

The Faculty of Health Sciences (FOHS) was established in 1974 with the opening of a medical school. Since the founding of the Medical School, additional schools have been added under the FOHS umbrella, including nursing, physiotherapy, medical laboratory sciences, paramedic training, a pharmacy program, and an English-language medical school for international students. In addition, the FOHS is currently running several postgraduate programs in public health, epidemiology, gerontology, health systems management, and basic medical sciences. In the academic year of 2015/2016 there were 2862 students in the various graduate programs, including 676 Israeli medical students in a regular program and 133 foreign medical students in an English-language program.

**Admissions:** Admission decisions are based on documentation of academic achievements and scores in the national psychometric tests. Applicants with the highest scores pass through a computerized sorting stage and then they are interviewed for the final selection process.

**Curriculum:** The Goldman School of Medicine aims to provide students with strong scientific background to foster their professional development as clinicians as well as researchers. Primary community medicine is strongly advocated. The first 2 years are devoted to courses in the basic sciences and early clinical exposure. The third year is devoted to the integration of basic sciences and clinical knowledge by integrative teaching of courses based on body systems. In the following 3 years students complete clinical rotations and electives, and prepare for their national and international qualifying examinations. Basic science, clinical skills, and behavioral sciences are taught in concert and are repeated over the years while adding levels of complexity to create meaningful clusters of knowledge, skills, and attitudes in the process of becoming a physician, i.e. a spiral curriculum [11]*.* Early exposure and learning in the community (about 20 % of curricular time) are emphasized [11]. Early clinical exposure starts from the first year.

**Faculty development:** The Office of Medical Education runs several programs and has a continual focus on faculty development [25]. Programs include a series of workshops and courses to build basic and advanced teaching skills for teachers in the sciences and for clinicians who teach at bedside. In recent years, workshops that aim to improve resilience and teach mindfulness methods have been introduced at various levels.

**Teaching methods:** The learning process includes lectures, small groups, self-directed learning, E-learning, modified PBL, CBL, and TBL. The flipped classroom method is used increasingly, and is highly recommended. Clinical skills are taught in pre-clerkship courses and in the clinical rotations. Simulation is used extensively.

**Infrastructure:** Facilities were originally built for lectures to large groups. As the teaching methodology shifted to small groups and e-learning, the campus has been adapted to provide teaching spaces for small groups and meeting rooms. The medical library provides teaching materials and supports a wide range of electronic teaching aids.

**Humanities:** The FOHS emphasizes development of professionalism through mandatory multi-annual courses in the humanities. These include literature and poetry in the first year, psychology and ethics in the second year, and philosophy and medical ethics in the third year. Additional humanities courses are Man and Society, and Becoming a Physician.

**Assessment:** Student performance is assessed primarily with MCQs during the preclinical years, with a combination of MCQs and extensive use of OSCEs during the clinical years. At the end of clinical rotations, students receive summative reports of their personal assessments.

**Promotion & Tenure:** Academic advancement for members of the medical faculty is based on a scoring system that takes into account scientific publications, teaching achievements, and leadership in clinical service.

**Service learning:** Students are encouraged to participate in community projects. The student union (ASRAN) is highly involved in the design and management of these activities. Some activities, for example workshops for high school students on safe and responsible sex, abstinence from alcohol, and reduction of violence are granted academic benefits. There are also projects for the children of Ethiopian immigrants, teaching first aid in the community, and teaching basic health concepts to kindergarten children.

**Recently added courses:** The newer additions to the medical curriculum include a unit in interprofessional education (IPE) where medical students learn together with nursing and physiotherapy students, and some of the humanities courses.

**Prizes & Citations:** Students take an active part in faculty evaluation. The best teachers and researchers are recognized and awarded a trophy in a special award ceremony at the end of the academic year.

**The Moshe Prywes Center for Medical Education:** Faculty development programs are led by the Moshe Prywes Center. These programs range from courses and workshops to counseling for individual teachers and course directors. Research activity is limited. Currently two PhD students are conducting research in medical education. The Center’s faculty currently includes a part-time senior physician with a background in medical education, and is currently aiming for expansion.

**Future plans:** Plans are underway to build a new simulation facility. In addition, the Faculty is in a process of a new strategic planning to re-evaluate its vision as well as its medical education efforts, and expects to redesign the curriculum for the coming 15 years.

### Faculty of Medicine in the Galilee, Bar Ilan University, Safed

This new school was established in 2011–2012 [1] and graduated its first class in 2015. The medical faculty was established in the city of Safed as part of the effort to strengthen the periphery of Israel, and specifically the Galilee [6]. The school’s graduate-level 4-year curriculum incorporates a 20-month clinical sciences phase and 2 years of clinical block rotations. An additional 3-year track offers clinical studies for Israeli medical students who have completed preclinical training outside of Israel. The Medical School currently has over 100 students for advanced degrees in the life sciences, including an MD-PhD program. Approximately 70 students are recruited annually for the 4-year MD program and 40 students per annum for the 3-year track. The faculty includes approximately 200 physicians with clinical academic appointments and 18 researchers in the preclinical faculty.

**Admissions:** Students are selected based on academic criteria followed by an MMI process in a format similar to that used at the Sackler Faculty of Medicine in MSR.

**Curriculum:** During the clinical sciences phase, students participate in five introductory block courses including public health, informatics, anatomy, basic pathology, and basic pharmacology. The introductory courses are followed by eight clinical fields designed with the innovative curriculum undertaken by the new medical school, integrating content around biological processes. The clinical fields currently include growth, reproduction, genetics and development, bio-energetics, immunity and transplantation, neoplasia, inflammation and infection, trauma, mind and brain, and aging and deterioration. Students also complete a longitudinal curriculum during the initial clinical sciences phase that includes an innovative mentoring program, a basic clinical skills course, and a medical humanities course. They then spend 2 years in clinical rotations. The medical school aims to reach 30 % ambulatory teaching in the future. Currently, 10–15 % of the teaching is community-based.

**Faculty development:** Three faculty development programs are underway: a clinical teacher training program based in affiliated hospitals, a preclinical course director program, a medical education foundation course for faculty. In addition, an anatomy teaching assistant training program has been implemented for selected students. A core group of faculty that shares a special interest in medical education has formed a learning community. This group meets regularly to review medical education literature and comprises an active research group involved in medical education research.

**Teaching methods:** While frontal lectures still dominate in the preclinical curriculum, the aim is to limit frontal teaching to a maximum of 50 %. Faculty development efforts are currently focused on encouraging small group teaching, including CBL and TBL. Course leaders are encouraged to decrease the proportion of frontal, passive teaching and to increase active, individualized and relevant teaching methods. In the clinical skills and humanities courses most of the teaching is in small groups. A preclinical doctoring course is comprised of weekly half-day meetings. The course takes place mainly in small groups and focuses not only on the basic skills of history taking and physical examination, but also on communication skills and other elements of professionalism. The course makes extensive use of standardized patients and simulators and incorporates evaluation by multiple OSCEs

**Infrastructure:** The new facility includes an auditorium and 12 classrooms suitable for small group teaching. A fully-equipped medical library, including extensive e-learning capabilities, is on-site.

**Humanities:** A longitudinal preclinical medical humanities program is incorporated into the medical sciences curriculum. This course is intertwined into the content of the entire clinical sciences phase and involves small-group work.

**Assessment:** Student performance evaluation relies primarily on MCQ-based examinations during preclinical studies, with use of OSCE and reflection-based evaluation in the clinical skills course. A new assessment tool, currently under development, will evaluate the school’s effectiveness in producing socially accountable graduates [26]. The teaching committee has formulated a list of primary learning outcomes defining the ideal graduate of the school.

**Promotion & Tenure:** Teaching and educational leadership is recognized and taken into consideration in academic promotion. There is no separate promotion track for teachers.

**Service learning:** The public health course includes a community involvement program that is assessed as an integral part of the course [26].

**Recently added courses:** A recent addition to the clinical curriculum is the ETGAR course that integrates students in the transition of patients from hospital to community care. This course involves ensuring patients’ understanding of the hospital discharge letter and home visits to facilitate continued treatment.

**Prizes & awards:** Excellent teachers and students are recognized annually by the Medical School and Bar Ilan University. Prizes are given for outstanding MD theses. No prizes for excellence in research exist yet.

**Office of Medical Education:** Staff of the Office of Medical Education includes a full-time PhD who heads assessment and promotion of teaching, and a part-time clinical faculty member focusing mainly on faculty development to improve clinical teaching. One part-time administrative assistant supports these two. Regular conferences and seminars are in place, including a weekly medical education Journal Club.

**Future plans:** The faculty plans to expand interprofessional education and increase the use of simulation.

## Abbreviations

BME: Basic Medical Education
CBL: Case-based learning
CHE: [Israeli] Council for Higher Education
CIP: Comprehensive Integrative Puzzle
ECFMG: [U.S.] Education Commission for Foreign Medical Graduates
FOHS: Faculty of Health Sciences
HEALER: Israeli Society for Medical Education
IMG: International Medical Graduate
IPE: Interprofessional Education
IRC: International Review Committee
MCQ: Multiple choice questions
MCSE: Multi Clinical Station Examination
MMI: Multiple mini-interviews
MOR: Hebrew acronym for ‘selection for Medicine’
MSR: National Simulation Center
NBME: [U.S.] National Board of Medical Examiners
OECD: Organization for Economic Cooperation and Development
OSCE: Objective structured clinical examination
PBL: Problem-based learning
TBL: Team-based learning
USMLE: U.S. Medical Licensing Examination
WFME: World Federation for Medical Education

## Footnotes

1 The three above cited committees came up with the range of 1000–1200 and not a specific fixed number, as needed to maintain the required level of physician to population ratio. We will use either this range or 1100 across the paper
